# Comparative analysis of the *Liriomyza chinensis* mitochondrial genome with other Agromyzids reveals conserved genome features

**DOI:** 10.1038/s41598-018-27213-7

**Published:** 2018-06-11

**Authors:** Jing-Yun Chen, Ya-Wen Chang, Si-Zhu Zheng, Ming-Xing Lu, Yu-Zhou Du

**Affiliations:** 1 School of Horticulture and Plant Protection & Institute of Applied Entomology, Yangzhou University, Yangzhou, 225009 China; 2 Suzhou Customs (formerly Suzhou Entry-Exit Inspection and Quarantine Bureau), Suzhou, 215000 China; 3 Joint International Research Laboratory of Agriculture and Agri-Product Safety, Yangzhou University, Yangzhou, 225009 China

## Abstract

*Liriomyza chinensis* is a serious pest of onions in many countries, especially in East Asia. We sequenced the complete mitochondrial genome of this species and compared it with five other Agromyzidae species. The *L*. *chinensis* mitogenome is a double-stranded 16,175 bp circular molecule with an A + T content of 78.3%. It contains 37 genes and a control region as do the sequenced *Liriomyza* species. The mitogenomes of *L*. *chinensis* and other Agromyzidae species showed a clear bias in nucleotide composition with a positive AT-skew. Most PCGs used standard ATN as start codons, and TAN as termination codons. The tRNAs exhibited the typical clover-leaf structure, except for tRNASer^(AGN)^ and the two rRNA genes are conserved with those of other Agromyzids. The *L*. *chinensis* mitogenome control region included several conserved regions, including a poly-T, two (TA)n and one poly-A stretch, which are considered important replication and transcription. The 13 PCGs were used to study the phylogeny of *L*. *chinensis* and five related Agromyzids. Analysis by maximum likelihood, Bayesian inference and genetic distance suggest congruent phylogenetic relationships in *Liriomyza* spp. and provide a useful supplement to taxonomic classification by morphology.

## Introduction

Mitochondria are involved in energy metabolism, apoptosis, aging, disease and oxidative phosphorylation^1–3^. Arthropod mitochondrial genomes (mtDNA) are generally circular, duplex molecules of 14–19 kb in length^4–6^. Insect mtDNA genomes contain a remarkably conserved set of 37 genes including 22 transfer RNA (tRNA) genes, two ribosomal RNA (rRNA) genes, 13 protein-coding genes (PCGs) and a control region (CR) or A + T-rich non-coding region^6,7^. Mitochondrial genomes have been widely used in phylogenetic studies and comparative and evolutionary genomics of insects and as molecular markers of population genetics and evolution^6,8–10^.

*Liriomyza chinensis* belongs to the group Phytomyzinae, family Agromyzidae and order Diptera and causes significant damage to *Allium* spp.^11^. The damage incited by *L*. *chinensis* on onions is very similar to other *Liriomyza* spp., the mining of leaves by larvae and puncturing of foliage by females for feeding and oviposition reduces photosynthesis, which leads to lower crop quality and quantity^12–15^. The leafminer *L*. *chinensis* has become a serious pest of onions in many countries and regions, especially in East Asia^11,16,17^. The taxonomic status of *L*. *chinensis* is particularly controversial over the past decades, but now its classification status is settled. Kato considered *L*. *chinensis* as a sub-species of *Dizygomyza cepae* (also known as *L*. *cepae*)^18^, and Hering reported that larvae of *L*. *chinensis* and *L*. *cepae* had identical spiracle structures. The male genitalia of *L*. *chinensis* show similarity to *L*. *cepae* and both species have characteristically pale wings and solid black scutellum^19^, these characteristics are different from the typical form of *Liriomyza*, represented by *L*. *nietzkei*^11^. Subsequent speciation produced *L*. *chinensis* in China, Japan and Malaysia, and *L*. *cepae* in western Europe, which are reproductively isolated from *L*. *nietzkei*. Consequently, Spencer upgraded the classification of *L*. *chinensis* from sub-species to species and assigned it to the genus *Liriomyza*^11^.

The systematics of Agromyzids is rather poorly understood due to their small size and morphological homogeneity. For molecular phylogenetic study of Agromyzids, Scheffer *et al*. investigated the phylogenetic relationships among genera within the Agromyzidae using parsimony and Bayesian analyses of the mitochondrial COI gene, the nuclear ribosomal 28 S gene, and the single copy nuclear CAD gene^20^. But the study on phylogenetic relationship of the genus *Liriomyza* based on whole mitochondrial genome was relatively limited. The development of improved sequencing technology has generated the complete or near complete mitogenomes of five Agromyzids including *L*. *sativae*^21^, *L*. *trifolii*^22,23^, *L*. *bryoniae*^23^, *L*. *huidobrensis*^24^, and *Chromatomyia horticola*^25^, which provide the basis for studying the phylogeny of Agromyzid species.

The mtDNA sequence of *L*. *chinensis* has not been previously reported and would be valuable in clarifying the taxonomic issues described above. In this paper, we report the complete mitochondrial genome of *L*. *chinensis* and provide a thorough description of its structural features. The *L*. *chinensis* mitogenome was compared with mtDNA sequences of five other related species to better understand taxonomy and phylogeny within the Agromyzidae.

## Results and Discussion

### Genome organization

**Figure 1:**
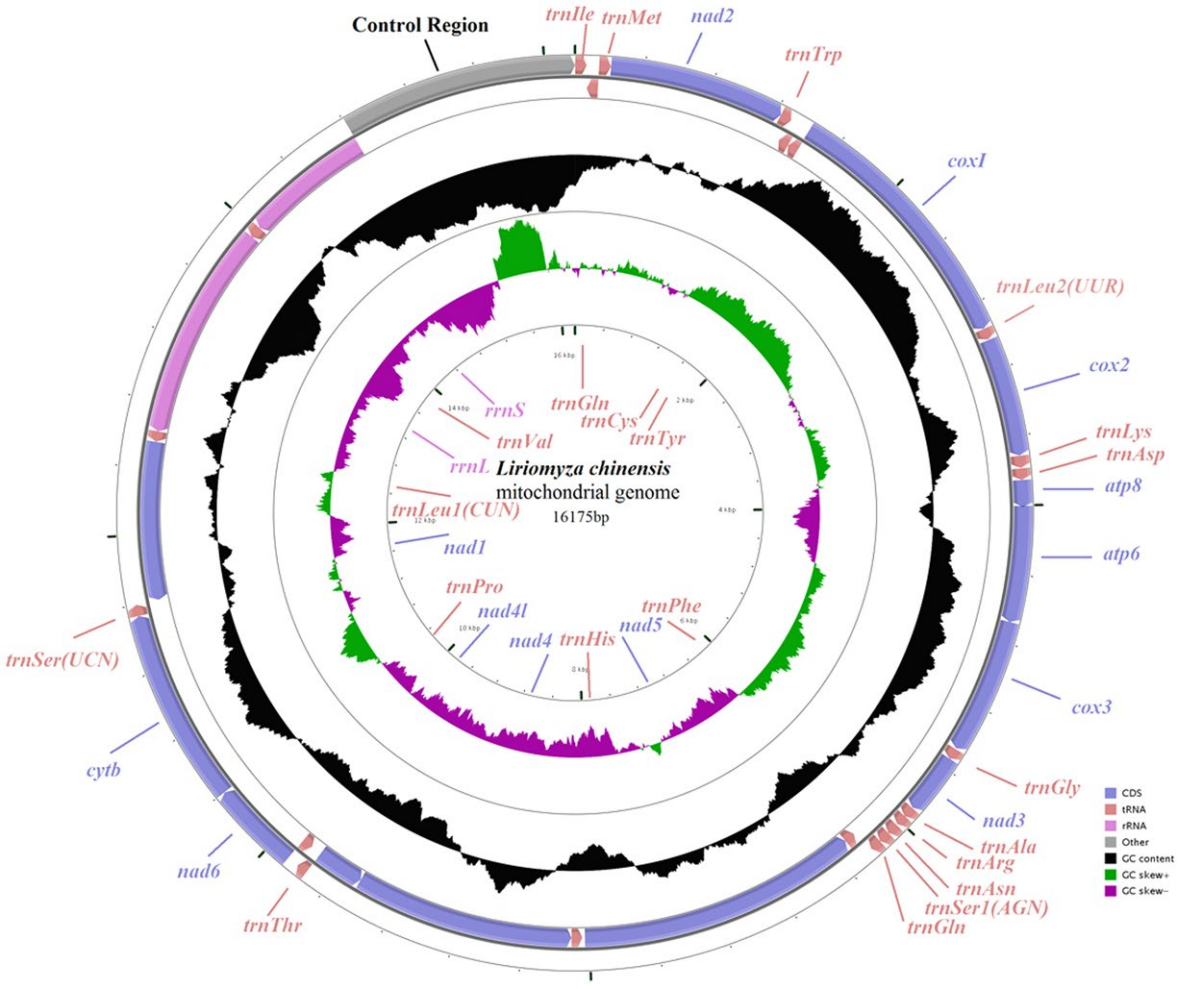
Map of mitochondrial genome of *L*. *chinensis*. Genes outside the map are transcribed in a clockwise direction (J-strand), whereas those inside the map are transcribed counterclockwise (N-strand). The second circle shows the GC content and the third shows the GC skew. GC content and GC skew are plotted as the deviation from the average value of the entire sequence.

The complete mitochondrial genome of *L*. *chinensis* is a circular 16,175 bp molecule (GenBank accession no. MG252777). It includes 37 mitochondrial genes (13 PCGs, 22 tRNA genes and two rRNA genes) and a large non-coding region (control region) (Fig. 1). The gene order in the *L*. *chinensis* mitochondrial genome is identical to *D*. *melanogaster*^26^, which is the classic structure for Diptera. There are 23 genes located on the J-strand (nine PCGs and 14 tRNAs) and 14 genes on the N-strand (four PCGs, eight tRNAs and two rRNAs). Sixteen intergenic spacers were identified with a total length of 64 bp; these ranged in size from 1–19 bp with the longest intergenic spacer located between tRNA^Glu^ and tRNA^Phe^. There were nine overlapping genes in the mitochondrial genome of *L*. *chinensis*; the longest overlap was 8 bp and mapped between tRNA^Trp^ and tRNA^Cys^ (Table 1).

**Table 1:** Annotation of the mitochondrial genome of *L*. *chinensis*.

Feature	Region		Direction	Length	Codon		Intergenic Nucleotides
	From	To			Start	Stop	
tRNA^Ile(I)^	1	67	F	67			
tRNA^Gln(Q)^	71	139	R	69			3
tRNA^Met(M)^	139	207	F	69			−1
*ND2*	208	1228	F	1021	ATT	T	−1
tRNA^Trp(W)^	1229	1295	F	67			0
tRNA^Cys(C)^	1288	1351	R	64			−8
tRNA^Tyr(Y)^	1353	1416	R	64			1
*COI*	1414	2953	F	1540	ATCA	TAA	−4
tRNA^Leu(UUR)^	2955	3020	F	66			1
*COII*	3024	3713	F	690	ATG	TAA	2
tRNA^Lys(K)^	3715	3785	F	71			1
tRNA^Asp(D)^	3788	3854	F	67			2
*ATP8*	3855	4010	F	156	ATT	TAA	0
*ATP6*	4004	4678	F	675	ATG	TAA	−7
*COIII*	4678	5469	F	792	ATG	TAA	−1
tRNA^Gly(G)^	5475	5539	F	65			5
*ND3*	5540	5893	F	354	ATT	TAA	−1
tRNA^Ala(A)^	5896	5959	F	64			2
tRNA^Arg(R)^	5960	6022	F	63			0
tRNA^Asn(N)^	6029	6094	F	66			6
tRNA^Ser(AGN)^	6095	6161	F	67			0
tRNA^Glu(E)^	6162	6228	F	67			0
tRNA^Phe(F)^	6248	6314	R	67			19
*ND5*	6315	8028	R	1714	ATT	T	−1
tRNA^His(H)^	8044	8109	R	66			15
*ND4*	8110	9448	R	1339	ATG	T	0
*ND4L*	9449	9738	R	290	ATG	TA	0
tRNA^Thr(T)^	9741	9804	F	64			2
tRNA^Pro(P)^	9805	9870	R	66			0
*ND6*	9873	10397	F	525	ATT	TAA	1
*CYTB*	10399	11533	F	1135	ATG	T	1
tRNA^Ser(UCN)^	11534	11599	F	66			0
*ND1*	11602	12564	R	963	GTG	TAG	2
tRNA^Leu(CUN)^	12566	12629	R	64			1
16 S rRNA	12630	13952	R	1323			0
tRNA^Val(V)^	13952	14023	R	72			−1
12 S rRNA	14024	14808	R	785			0
control region	14809	16175		1367			0

The mitochondrial genome length of *L*. *chinensis* was similar to other Agromyzidae family members. Exceptions were *L*. *sativae* and *C*. *horticola*; the former contains a short A + T region^21^, and the latter lacks an A + T region due to incomplete sequencing^25^. The gene order is identical to the ancestral *trnI*–*trnQ*-*trnM* arrangement. There were no gene rearrangements in the six Agromyzidae mitogenomes, which indicates that the mitochondrial gene order is highly conserved in Agromyzidae. Furthermore, the length and position of intergenic spacers was also highly conserved.

### Nucleotide Composition

The nucleotide composition of the *L*. *chinensis* mtDNA showed an obvious bias for A and T. The A + T content of the whole genome was 78.3% (A = 41.3%, T = 37.0%, G = 8.9%, C = 12.8%). The A + T content of isolated PCGs, tRNAs, rRNAs, and the control region exceeded 75%, and the control region had the highest A + T content (89.4%) (Table 2). This strand bias in nucleotide composition is a universal phenomenon in metazoan mitochondrial genomes and is evident by a comparative analysis of AT- and GC-skews^4,6,27^. The Agromyzids mtDNAs showed a positive AT- and negative GC-skew over the entire genome (Table 3). The PCGs, tRNAs and rRNAs of the six Agromyzids mtDNAs show a relatively consistent A + T content and AT-skew (Table 3). The A + T-rich region *L*. *chinensis* exhibited a lower A + T content (~90%) in comparison to the other congener mitogenomes. The underlying mechanism of the A + T bias has been explained by asymmetric mutation and selection pressure during replication and transcription^28^. We tried to determine if there were any relationships between the A + T content and phylogeny but clear patterns were not evident. The A + T nucleotide bias has significance for the study of replication, transcription and rearrangement of the mitochondrial genome.

**Table 2:** Nucleotide composition of the *L*. *chinensis* mitogenome in different regions.

Region	Nucleotides Proportions (%)						AT-skew	GC-skew
	A	T	G	C	A + T	G + C		
Whole genome	41.3	37.0	8.9	12.8	78.3	21.7	0.05	−0.18
Protein coding genes	40.1	36.0	9.9	14.0	76.1	23.9	0.05	−0.17
1st codon position	40.0	34.7	11.8	13.5	74.7	25.3	0.07	−0.07
2nd codon position	36.7	35.6	10.7	17.0	72.3	27.7	0.02	−0.23
3rd codon position	43.8	37.6	7.1	11.5	81.4	18.6	0.08	−0.24
tRNA genes	40.3	37.3	10.1	12.2	77.6	22.3	0.04	−0.09
16 S rRNA	43.8	39.5	5.9	10.8	83.3	16.7	0.05	−0.29
12 S rRNA	41.4	39.6	6.9	12.1	81.0	19.0	0.02	−0.27
control region	49.2	40.2	4.5	6.1	89.4	10.6	0.10	−0.15

**Table 3:** Nucleotide composition in regions of Agromyzidae mitogenomes.

Species	Whole	PCGs	tRNA	rRNA	control region	AT-skew	GC-skew
	A + T%	A + T%	A + T%	A + T%	A + T%		
*L*. *sativae*	77.5	75.7	77.1	82.2	93.0	0.05	−0.18
*L*. *trifolii*	78.2	75.6	77.2	82.6	93.6	0.05	−0.19
*L*. *huidobrensis*	78.3	75.7	77.4	82.2	93.0	0.04	−0.17
*L*. *bryoniae*	79.3	76.7	78.5	82.4	95.5	0.04	−0.19
*C*. *horticola*	78.2	76.1	76.9	81.4	N/A	0.05	−0.16

N/A: data not available.

### Protein-coding genes

The nucleotide bias was also reflected in the 13 PCGs, which had a relatively high A + T percentage (~76.1%, Table 2). The average A + T content among PCGs in *L*. *chinensis* was 76.1%. The A + T content of the third codon position (81.4%) was higher than the first (74.7%) and second codon (72.3%) positions (Table 2), which may suggest that both higher mutation rates and increased A + T content are related and depend on a relaxed selection at the third codon position^21,29^.

Eleven PCGs of *L*. *chinensis* were found to initiate with ATN (five with ATT and six with ATG). However, *ND1* and *COI* started with GTG and the special quadruplet start codon of ATCA (Table 1), respectively, which agreed with *L*. *trifolii* and *L*. *sativae*, but differed from other Agromyzidae species^10,21^. These special start codons are converted into typical initiation codons by RNA editing during transcription^30^, which can reduce the intergenic spacer and avoid gene overlap^31^.*COI* genes generally use nonstandard and varied start codons in insects. Among the six Agromyzids, five *Liriomyza* species all used “ATCA” as special quadruplet start codon^21,23^, while for *C*. *horticola*, which used the “TTG” as nonstandard start codon^25^. Consequently, the use of nonstandard initiation codon in *COI* gene was not unexpected and it was shown to be dependent on the translated amino acid sequence and subsequent sequence alignments^32^. Eight PCGs used the typical termination codons TAA and TAG (*ND1*), whereas *ND2*, *ND5*, *ND4*, and *CYTB* used incomplete stop codons with T as a termination signal (Table 1). *ND4L* used TA as a termination signal, which has been reported in other Agromyzids^10^. Incomplete termination codons are common in metazoan mitochondrial genomes. It has been speculated that the polyadenylation site is generated by adding A to the 3’ end of the mRNA transcript, which is then converted into a complete stop codon for termination of transcription^33^.

**Figure 2:**
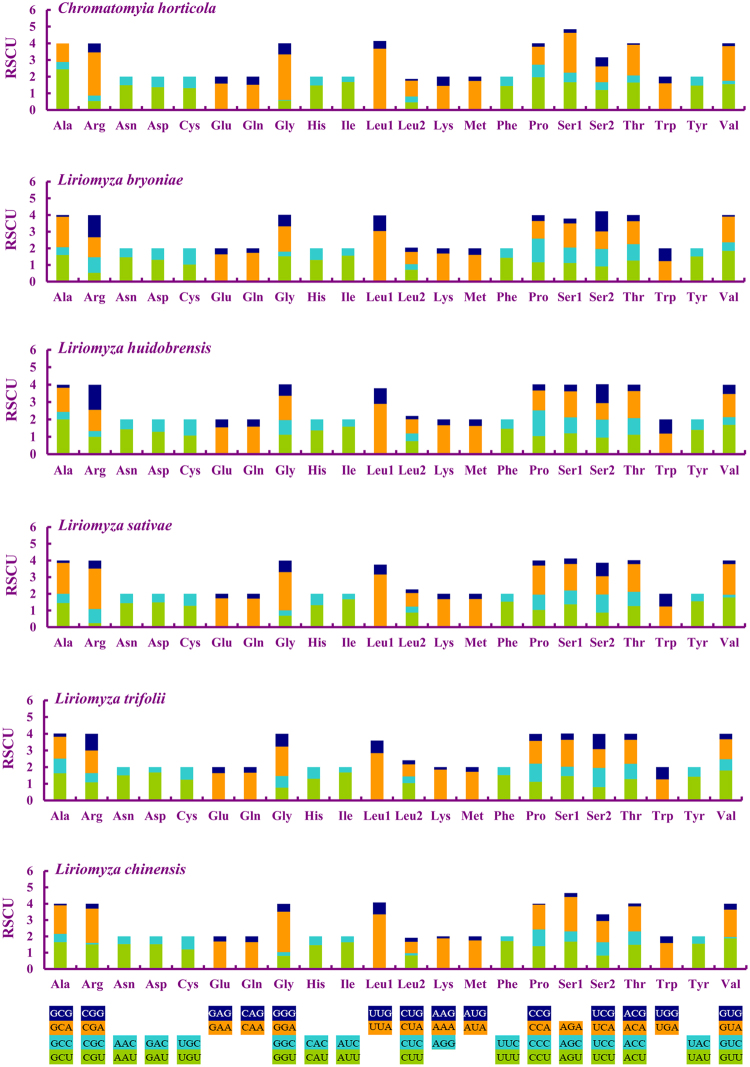
The mitochondrial genome relative synonymous codon usage (RSCU) across six Agromyzidae flies. Codon families are provided on the X axis.

The relative synonymous codon usage (RSCU) values of the *L*. *chinensis* mitogenome were calculated and illustrated, and the RSCU for Agromyzidae is shown (Fig. 2). The use of anticodons NNA and NNU indicated a preference for A or T in the third nucleotide of PCG anticodons. All possible codons are present in the PCGs of *L*. *chinensis* and the other four *Liriomyza* spp., whereas GCG was not found in *C*. *horticola*. Previous research indicates that codons with high G and C content are generally not favored, a phenomenon with low GC content that is found in some insects, such as moths^34,35^, stonefly^36^, whitefly^37^ etc.

**Figure 3:**
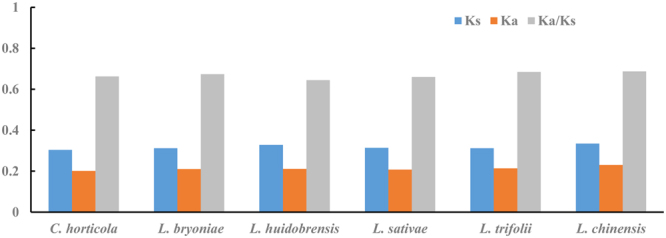
Evolutionary rates of Agromyzidae flies mitochondrial genomes. The number of nonsynonymous substitutions per nonsynonymous site (Ka), the number of synonymous substitutions per synonymous site (Ks), and the ratio of Ka/Ks for each Agromyzidae flies mitochondrial genome are given, using that of *D*. *melanogaster* as a reference sequence.

The rates of nonsynonymous (Ka) and synonymous substitutions (Ks) and the Ka/Ks ratio were calculated for all PCGs in the six Agromyzidae mtDNA genomes using *D*. *melanogast*e*r* as a reference sequence (Fig. 3). All Ka values were less than Ks values; consequently, the Ka/Ks ratios were less than 1 (Fig. 3), indicating the likelihood of purifying selection in these species^38^. Agromyzidae species generally show relatively consistent evolutionary rates, which may be related to their relatively constant habitat as larval leafminers^39,40^.

### tRNA genes

**Figure 4:**
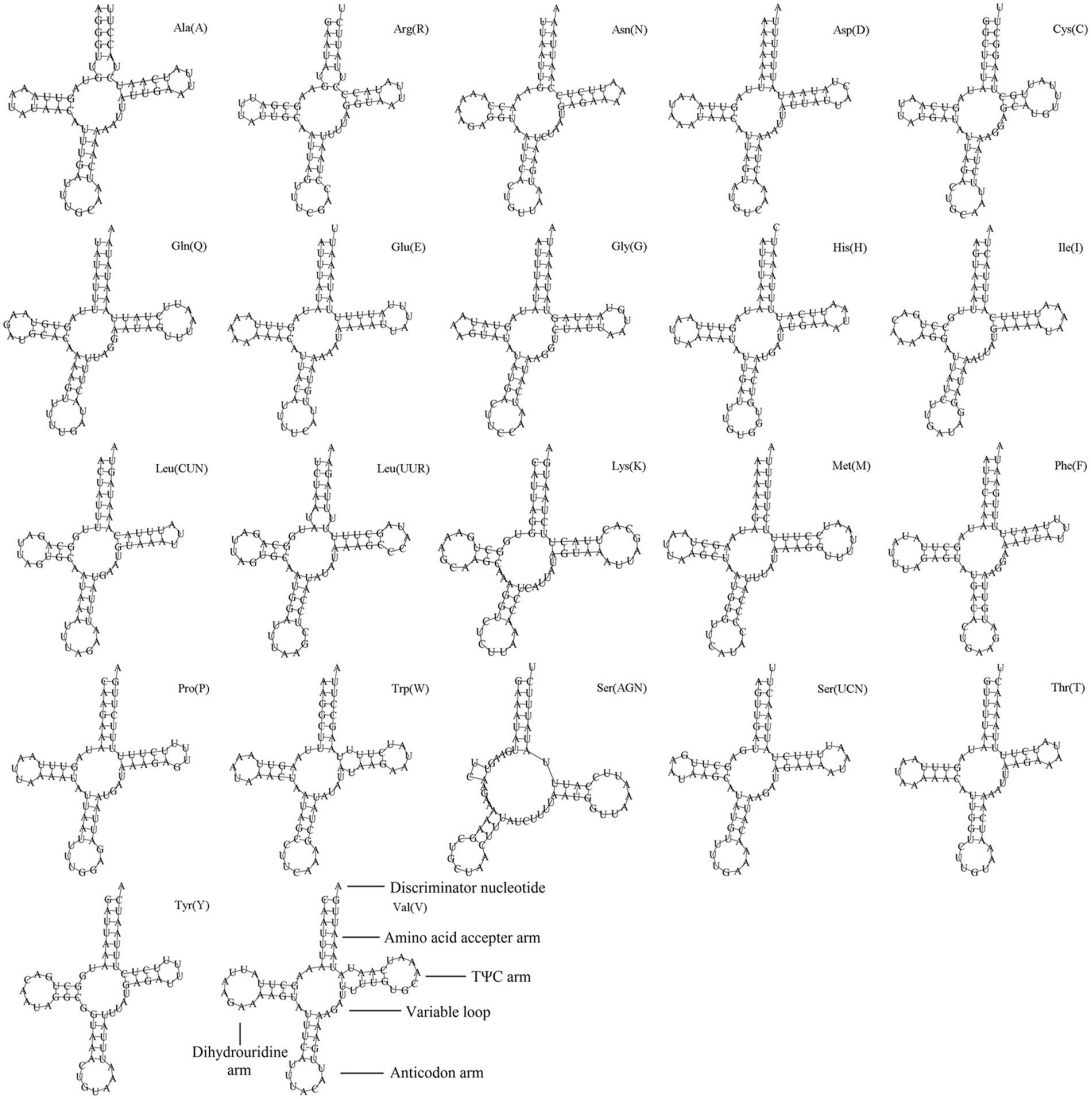
Inferred secondary structures of tRNAs from the *L*. *chinensis* mitogenome. The tRNAs are labelled with the abbreviations of their corresponding amino acids. Structural elements in tRNA arms and loops are illustrated as for *trnV*.

The tRNA genes of all the six Agromyzidae species contained an A + T content exceeding 76%. Twenty-two complete tRNAs were identified in the *L*. *chinensis* mtDNA, and 20 were discovered using tRNAscan-SE. tRNA^Arg^ and tRNA^Ser(AGN)^ could not be detected by software, but were instead determined through comparison with published Agromyzidae mitochondrial genomes (Fig. 4). The typical number of tRNA genes in the mtDNA of Agromyzids was 22, but in *L*. *trifolii* and *L*. *bryoniae*, there were two additional tRNA genes in the A + T-rich region near 12 S rRNA^10^. Since the anticodons of the four additional tRNAs were atypical, Yang *et al*. suggested that these additional tRNAs were generated by gene duplications that could be folded into tRNA-like secondary structures but were nonfunctional^10^. All *L*. *chinensis* tRNAs folded into the typical clover-leaf structure except for tRNA^Ser(AGN)^, which lacked the dihydrouridine (DHU) arm (Fig. 4). The DHU arm of tRNA^Ser(AGN)^ formed a large loop instead of the conserved stem-and-loop structure (Fig. 4). Atypical numbers and structures of tRNA have been reported in other insects^8,38,41–43^. The factors that may have led to these truncated tRNAs remain unknown, although truncation may be a result of generalized evolutionary pressures for size reduction in mitogenomes^6,44^, but such an explanation requires the existence of compensatory mechanisms. Thus, the abnormal structure of tRNA^Ser(AGN)^ warrants further study. The *L*. *chinensis* tRNAs ranged from 63 (tRNA^Arg(R)^) to 72 (tRNA^Val(V)^) nucleotides; the length of the tRNA usually depends on the size of the variable and D-loops^45^. Based on the secondary structure of the tRNAs in the *L*. *chinensis* mtDNA, there were 19 unmatched nucleotides including 14 G-U and five U-U pairs; these mapped to the amino acid, TψC, and anticodon arms. A total of 27 mismatched bases were reported in the tRNA of *L*. *sativae*, these included 21 G-U, four U-U, one A-A and one A-C pairs that were located in the AA (8 bp), DHU (10 bp), AC (5 bp) and TψC arms (4 bp), respectively^21^.

### rRNA genes

The boundaries of rRNA genes were identified by sequence alignment with published Dipteran sequences. The *L*. *chinensis* mtDNA contained the 16 S rRNA and 12 S rRNA, which mapped between tRNA^Leu(CUN)^/tRNA^Val^ and tRNA^Val^/control region, respectively (Fig. 1). The 16 S and 12 S rRNA genes are 1323 and 785 bp with an A + T content of 83.3 and 81.0%, respectively. The two rRNAs mapped to the same location as described for other Agromyzidae mitogenomes. Both the 16 S and 12 S rRNAs have been widely used for population genetics, molecular phylogeny and species identification^46,47^. However, the rRNA sequences of Agromyzids were relatively conserved^23^, and thus would not provide much useful insight regarding population genetics. However, the secondary structure of rRNA genes in *Liriomyza* spp. may contain potentially useful information. For example, the 12 S rRNA of *L*. *huidobrensis* showed more variability in sequence and structure of the H51-H100 region arm as compared to *L*. *trifolii*; thus, this region may be a potential marker for identification of *Liriomyza* spp.^24^.

### Control Region (A + T-rich region)

**Figure 5:**
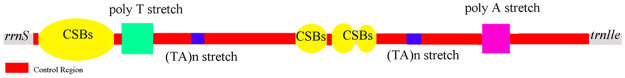
Predicted structural elements in the control region of *L*. *chinensis*. The genes flanking the control region, 12 S RNA and tRNA^Ile(I)^, are represented in gray boxes; the red-shaded rectangles indicate A + T-rich regions; the purple/green box indicates conserved poly A/T structures; yellow boxes indicate conserved sequence blocks with other leafminer species; and blue box indicates (TA) n stretches by using the Tandem Repeats.

The control region of the *L*. *chinensis* mtDNA is located between the 12 S rRNA and tRNA^Ile^ genes (Fig. 1); it consists of 1367 nucleotides and has the highest A + T content (89.4%) of the mtDNA genome (Table 1). This region varies greatly in length among insects, ranging from 70 bp to 13 kb^48,49^, and it accounts for most of the variation in mtDNA size. The control regions in *L*. *trifolii*, *L*. *bryoniae*, *L*. *huidobrensis*, and *L*. *sativae* have a high A + T content (>90%), also map between the 12 S rRNA and tRNA^Ile^ genes, and are 1338, 1354, 1416, and 741 bp in length, respectively^21,22^. The A + T-rich region is the fastest evolving region in the mitochondrial genome^50^, and a comparative analysis of mtDNA sequences in *Drosophila* shows that divergence in the control region is very significant in most species^51^. However, there are five conserved structural elements have been found in the control region of many insects including a poly-T stretch, a [TA(A)]n-like stretch, a highly conserved stem-and-loop structure, a pair of sequences immediately flanking the stem/loop structure with reiterated TATA and G(A)n T consensus sequences, and a G + A-rich stretch downstream of the secondary structure^49^. We identified several conserved structural elements in the control region of the *L*. *chinensis* mtDNA; these included one poly-T stretch, two (TA)n stretches and one poly-A stretch (Fig. 5). The A + T-rich region is the largest noncoding region in mtDNA and is associated with replication and transcription, which is why it is named the control region^6,52^. It is highly variable both in content and size due to insertions and deletions, variation in copy numbers of tandem repeats, and extensive change in the length of a variable domain^50,53,54^. Studies have shown that the A + T-rich region harbors sufficient polymorphisms to be a suitable marker for studying population genetics and phylogenetic reconstruction of closely related taxa^55,56^.

### Phylogenetic analysis

**Figure 6:**
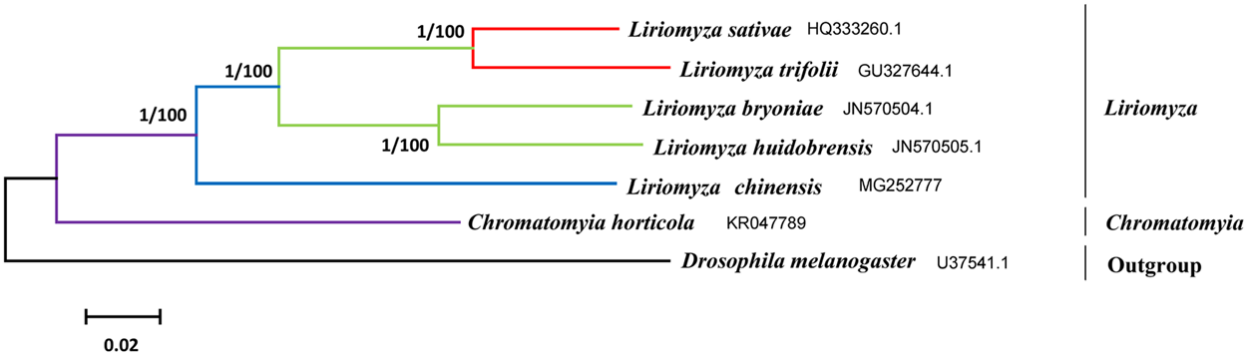
Inferred phylogenetic relationships among Agromyzidae based on nucleotide sequences of 13 protein-coding genes using Bayesian inference (BI) and maximum likelihood (ML). Numbers at each node indicate bootstrap support; percentages of ML bootstrap support values (first value) and Bayesian posterior probabilities (second value), respectively. *D*. *melanogaster* was used as an outgroup^59^. The scale bar indicates the number of substitutions per site.

We performed phylogenetic analysis of mtDNA using the nucleotide sequences of 13 PCGs in six Agromyzid mitochondrial genome sequences; *D*. *melanogaster* served as an outgroup. The topology of two phylogenetic trees constructed separately by maximum likelihood (ML) and Bayesian inference (BI) analyses were very similar. *L*. *sativae* grouped with *L*. *trifolii*, while *L*. *huidobrensis* and *L*. *bryoniae* were in another group, and *L*. *chinensis* was situated between the other *Liriomyza* spp. and *Chromatomyia* (Fig. 6). Phylogenetic analyses indicated that *L*. *trifolii*, *L*. *sativae*, *L*. *huidobrensis* and *L*. *bryoniae* are closely related; however, it was difficult to determine which *Liriomyza* spp. was most closely related to *L*. *chinensis* (Fig. 6).

Interspecific divergence spanned from 8.5% (*L*. *sativae* and *L*. *trifolii*) to 20.8% (*C*. *horticola* and *L*. *chinensis*) (Table 4). The genetic distance between DNA sequences is an important characteristic for classification and identification^57^, and a 2% genetic distance was previously proposed as the threshold between species^58^. In this study, the genetic distance far exceeded 2%, which is consistent with the classification of the six Agromyzids as distinct species. The genetic distance among *Liriomyza* spp. was close to each other, while the genetic distance between *C*. *horticola* and other *Liriomyza* species was far away. In general, the pattern of genetic distance was consistent with the phylogenetic tree. During the long-term evolution progresses, the highly invasive polyphagous species (such as *L*. *trifolii*, *L*. *sativae*, *L*. *huidobrensis* and *L*. *bryoniae*) have similar host niches and environmental stress, so the genetic distance was close to each other. The convergence of environmental variation and ecological factors can influence speciation^59^; however, the genetic distance between *L*. *chinensis* and other *Liriomyza* species was relatively far, which probably resulted from the substantial differences in food preferences of *L*. *chinensis*^60^.

**Table 4:** Interspecies average divergence of Agromyzidae based on the Kimura-2-parameters model.

	*L*. *bryoniae*	*L*. *huidobrensis*	*L*. *sativae*	*L*. *trifolii*	*L*. *chinensis*	*C*. *horticola*
*L*. *bryoniae*		0.096	0.147	0.156	0.179	0.189
*L*. *huidobrensis*	0.096		0.145	0.157	0.186	0.192
*L*. *sativae*	0.147	0.145		0.085	0.182	0.188
*L*. *trifolii*	0.156	0.157	0.085		0.183	0.194
*L*. *chinensis*	0.179	0.186	0.182	0.183		0.208
*C*. *horticola*	0.189	0.192	0.188	0.194	0.208	

Based on our data, the genus *Liriomyza* has relatively conserved mtDNA genomes and phylogenetic relationships, which conform the assignment of *L*. *chinensis* to the genus *Liriomyza* and provide a useful supplement to traditional taxonomic classification.

## Materials and Methods

### Sample and DNA extraction

Specimens of *L*. *chinensis* were collected from onions at Laiwu (36.12°N, 117.04°E) in Shangdong, China. All specimens were preserved in 100% ethanol and stored at -20 °C until DNA extraction was performed. Genomic DNA was extracted from samples using AxyPrep^TM^ Multisource Genomic DNA Kit (Axygen, California, USA) and then used for PCR.

### PCR amplification and sequencing

The mitochondrial genome of *L*. *chinensis* was amplified from extracted genomic DNA using short, overlapping PCR fragments (<1.2 kb). Twenty-five universal primer pairs specific for Diptera mtDNA^61^ were designed using Primer Premier 5.0 software (Supplementary Table S1). Conditions for PCR amplification were as follows: initial denaturation for 5 min at 94 °C, followed by 35 cycles of denaturation for 1 min at 94 °C, annealing for 1 min at 45–55 °C, elongation for 1.5 min at 72 °C, and a final extension step of 72 °C for 10 min. These PCR products were analyzed by 1.0% agarose gel electrophoresis and purified with an Axygen DNA Gel Extraction Kit (Axygen Biotechnology, Hangzhou, China). All amplified products were sequenced in both directions. If the sequenced result was bimodal, fragments were cloned into pGEM-T easy and re-was sequenced after cloning.

### Sequence Assembly, Annotation and Analysis

Protein-coding genes (PCGs) and rRNA genes in the *L*. *chinensis* mtDNA were identified by comparative analysis with other Agromyzidae family members. PCGs were aligned using Clustal X version 2.0^62^ and the boundaries of individual genes were confirmed with ORF finder (https://www.ncbi.nlm.nih.gov/orffinder/). The mitogenomic map was depicted with CG View Server (http://stothard.afns.ualberta.ca/cgview_server/), and PCG nucleotide sequences (lacking start and termination codons) were translated using MEGA v. 6.0^63^. Both the A + T content and codon usage were calculated using MEGA v. 6.0. Skew analysis was carried out with formulas AT-skew = [A−T]/[A + T] and GC-skew = [G−C]/[G + C]^64^. The software package DnaSP v. 5.10^65^ was used to calculate synonymous substitution (Ks) and nonsynonymous substitution rates (Ka). Most tRNAs were recognized by tRNAscan-SE v. 1.21 (http://lowelab.ucsc.edu/tRNAscan-SE/), and tRNAs that could not be identified using tRNAscan-SE were confirmed by sequence comparison with other Dipteran insects^66^. The tandem repeats in the putative control region were analyzed with Tandem Repeats Finder (http://tandem.bu.edu/trf/trf.advanced.submit.html).

### Phylogenetic Analysis

Phylogenetic analyses were based on nucleotide sequence of 13 PCGs derived from *L*. *chinensis* and five other Agromyzidae - *L*. *sativae*, *L*. *trifolii*, *L*. *bryoniae*, *L*. *huidobrensis*, and *C*. *horticola* mitogenomes available from GenBank (GenBank accession nos. HQ333260.1, GU327644.1, JN570504.1, JN570505.1 and KR047789, respectively). The mitogenome of *Drosophila melanogaster* (U37541.1) was used as the outgroup^26^. The nucleotide sequences of the 13 PCGs were initially aligned with Clustal X, translated into amino acids using default settings, and then analyzed with MEGA v. 6.0. Alignments of individual genes were concatenated using default settings, and the stop codon was excluded. Phylogenetic analysis was conducted using maximum likelihood (ML) and Bayesian inference (BI), which were conducted with MEGA v. 6.0 and MrBayes v. 3.1.2^67^. The ML method was used to infer phylogenetic trees with 1000 bootstrap replicates. BI analyses were conducted under the following conditions: 1,000,000 generations, four chains (one cold chain and three hot chains) and a burn-in step for the first 10,000 generations. The confidence values of the BI tree were expressed as Bayesian posterior probabilities in percentages. Simultaneously, interspecific genetic divergence was calculated by MEGA v. 6.0 using the Kimmura-2-parameter model^68^.

### Accession codes

Sequence data used in this study was deposited in GenBank (accession number MG252777).

## Electronic supplementary material

Supplementary Table S1
