# Supplementary material for: Comparative analysis of the Liriomyza chinensis mitochondrial genome with other Agromyzids reveals conserved genome features
Source: Sci Rep. 2018 Jun 11;8:8850. doi: 10.1038/s41598-018-27213-7 (PMC5995824; doi:10.1038/s41598-018-27213-7)
Supplement: Supplementary file 1 — Supplementary Table S1 [file 41598_2018_27213_MOESM1_ESM.pdf]

Comparative analysis of the *Liriomyza chinensis* mitochondrial genome with other Agromyzids reveals conserved genome features and phylogenetic relationships

Jing-Yun Chen, Ya-Wen Chang, Si-Zhu Zheng, Ming-Xing Lu, Yu-Zhou Du

Supplementary Table S1. List of PCR primers for *Liriomyza chinensis* mtDNA complete genome

Regions	Primer pair	Forward (5′-3′)	Reverse (5′-3′)
tRNA$^{Ile(I)}$ -*COI*	*Lc*mtDNA-01 (7-726)	AGTAAATTGCCTGATAAAAGG	TTGAWGAATAAGCTATTAATTT
	*Lc*mtDNA-02 (301-1444)	CTTGAATAGGATTAGAAATTA	AATAATCATTGTCGCAATGAT
	*Lc*mtDNA-03 (978-1835)	TTAGGRGGAYTRCCYCCATT	CCAGCTCCATTTTCWACTAT
	*Lc*mtDNA-04 (1494-2553)	TGAGCWGGAATAGTRGGAACTTC	GCTCCTATTGATARTACATAATG
	*Lc*mtDNA-05 (2212-2978)	CCHGGATTYGGWATAATYTCT	CTATGTTCAGCTGGYGGAGTA
COI-tRNA$^{Arg(R)}$	*Lc*mtDNA-06 (2637-3590)	AGCMGGATTTATTCAYTGAT	CTCCTAAAGCWGGKAYTGTT
	*Lc*mtDNA-07 (3311-4461)	GCTTTTCCHTCTYTWCGAYT	TTCCTTGAGGAACTAAATGA
	*Lc*mtDNA-08 (4076-4929)	ATTTTCAGTATTTGACCCYTC	TCTCGWGAWACATCTCGTCAT
	*Lc*mtDNA-09 (4518-5523)	CGACCWGGWACWTTAGCWGT	TAYCCTCCTCATCARTAAAT
	*Lc*mtDNA-10 (5206-6001)	TTCACAAACTACYCAAGGWTT	GTTATAWTTAACTACAACCC
ND3-ND4	*Lc*mtDNA-11 (5677-6496)	CCHTTYGAATGYGGATTTGA	GTWAAAARTTTTGATCAGGG
	*Lc*mtDNA-12 (6354-7369)	ATTTCCHTAACATCTTCARTG	ATTTATAGCTGGATTAGGRGC
	*Lc*mtDNA-13 (6999-7789)	TCGAAAWGAATAACWAACWGT	AGATGGWTTAGGAYTTGTT
	*Lc*mtDNA-14 (7512-8476)	TAGCWGCWGGTAATCAAGA	GCTCCTCCWACWTTRAATT
	*Lc*mtDNA-15 (8158-9156)	TCATATCAYTRACACCACA	GAGGKTATCARCCWGAACG

ND4-16S rRNA	*Lc*mtDNA-16 (8893-9876)	TTATAGAMCCAGAAACWGG	TTGRTTTACAAGACCAATG
	*Lc*mtDNA-17 (9620-10813)	GCHCCTTCACAWACTCTAAAWGT	CRTAATAWATTCCTCGTCCTA
	*Lc*mtDNA-18 (10599-11536)	TGGWTCATTAYTWGGATTAT	GTTCTTCWACTGGTCGWGCT
	*Lc*mtDNA-19 (11268-12253)	TATYCCWGCTAAYCCWTTAG	GGTTTRCGRGCTGTRGCTCA
	*Lc*mtDNA-20 (11931-12646)	CTTCTATATTCTACATTAAA	ACTATTTTGGCAGATTAGTGCAA
ND1- tRNA$^{Ile(I)}$	*Lc*mtDNA-21 (12286-13316)	AACCAGCWAYTATWACWGTAT	AGTATTTTRACTGTGCAAAGG
	*Lc*mtDNA-22 (13152-14217)	ATYTATAGGGTCTTCTCGTCT	AATATGYACACATCGCCCGTC
	*Lc*mtDNA-23 (13933-14590)	CTTTYACAATACTAATWMAC	WAAACTAGGATTAGATACC
	*Lc*mtDNA-24 (14197-14725)	GACGGGCGATRTGTRCATA	CCAGCAGTCGCGGTTATAC
	*Lc*mtDNA-25 (14670-29)	GCGGCTGCTGGCACAAATT	ATTTATCCTATCAAGATAA
